# Cost-effectiveness of an exercise program during pregnancy to prevent gestational diabetes: Results of an economic evaluation alongside a randomised controlled trial

**DOI:** 10.1186/1471-2393-12-64

**Published:** 2012-07-04

**Authors:** Nicolette Oostdam, Judith Bosmans, Maurice GAJ Wouters, Elisabeth MW Eekhoff, Willem van Mechelen, Mireille NM van Poppel

**Affiliations:** 1 Department of Public and Occupational Health, EMGO+ Institute for Health and Care Research, VU University Medical Center, Amsterdam, the Netherlands; 2 Department of Health Sciences and EMGO Institute for Health and Care Research, Faculty of Earth and Life Sciences, VU University Amsterdam, Amsterdam, the Netherlands; 3 Department of Obstetrics and Gynaecology, VU University Medical Center, Amsterdam, the Netherlands; 4 Department of Endocrinology, VU University Medical Center, Amsterdam, the Netherlands; 5 Body@Work, Research Center Physical Activity, Work and Health, TNO-VU University Medical Center, Amsterdam and Hoofddorp, the Netherlands; 6 Department of Public and Occupational Health, VU University Medical Center, Van der Boechorststraat 7, Room-C568, 1081 BT, Amsterdam, the Netherlands

**Keywords:** Cost-effectiveness, Pregnancy, Exercise

## Abstract

**Background:**

The prevalence of gestational diabetes mellitus (GDM) is increasing worldwide. GDM and the risks associated with GDM lead to increased health care costs and losses in productivity. The objective of this study is to evaluate whether the FitFor2 exercise program during pregnancy is cost-effective from a societal perspective as compared to standard care.

**Methods:**

A randomised controlled trial (RCT) and simultaneous economic evaluation of the FitFor2 program were conducted. Pregnant women at risk for GDM were randomised to an exercise program to prevent high maternal blood glucose (n = 62) or to standard care (n = 59). The exercise program consisted of two sessions of aerobic and strengthening exercises per week. Clinical outcome measures were maternal fasting blood glucose levels, insulin sensitivity and infant birth weight. Quality of life was measured using the EuroQol 5-D and quality-adjusted life-years (QALYs) were calculated. Resource utilization and sick leave data were collected by questionnaires. Data were analysed according to the intention-to-treat principle. Missing data were imputed using multiple imputations. Bootstrapping techniques estimated the uncertainty surrounding the cost differences and incremental cost-effectiveness ratios.

**Results:**

There were no statistically significant differences in any outcome measure. During pregnancy, total health care costs and costs of productivity losses were statistically non-significant (mean difference €1308; 95%CI €-229 - €3204). The cost-effectiveness analyses showed that the exercise program was not cost-effective in comparison to the control group for blood glucose levels, insulin sensitivity, infant birth weight or QALYs.

**Conclusion:**

The twice-weekly exercise program for pregnant women at risk for GDM evaluated in the present study was not cost-effective compared to standard care. Based on these results, implementation of this exercise program for the prevention of GDM cannot be recommended.

**Trial registration:**

NTR1139

## Background

The prevalence of gestational diabetes mellitus (GDM) is increasing worldwide [1], paralleling the increase in type 2 diabetes (T2D) [2]. Approximately 1-14% of pregnant women will develop GDM, depending on the population and the diagnostic procedure [3]. Risk factors for GDM are obesity (BMI ≥ 25), personal history of GDM, glycosuria, and/or a strong family history of diabetes. The presence of GDM is associated with an increased maternal risk for other pregnancy-related complications (preeclampsia, postpartum haemorrhage, caesarean section), and with an increased risk for developing T2D after pregnancy [4]. GDM also puts the infant at risk, since it is associated with an increased risk for macrosomia, jaundice and birth trauma [4]. Later in life, children whose mothers had GDM, have an increased risk of obesity, abnormal glucose tolerance, and T2D [4,5]. GDM and the risks associated with GDM lead to increased health care costs and productivity losses [6]. Therefore, the prevention of high blood glucose during pregnancy has become an important goal.

To prevent high blood glucose during pregnancy, there are various preventive strategies available, such as reducing a mother’s overweight and obesity, limiting gestational weight gain, improving diet and exercise behaviour as well as various drug therapies with glucose-lowering agents [7-10].

The effects of an exercise program (FitFor2) during pregnancy for women at risk for GDM were evaluated in a randomised controlled trial and have been reported elsewhere [11]. In short, no statistically significant effects of the intervention were found on maternal glucose, insulin sensitivity or the infant’s birth weight compared to the control group. However, although there were no effects on clinical outcomes, the FitFor2 exercise program may be a cost-effective intervention to prevent high maternal blood glucose during pregnancy. To our knowledge, no studies have been published on the cost-effectiveness (CE) of such an intervention; therefore, in this paper we report on the cost-effectiveness the program in comparison to standard care.

## Methods

### Study design

The economic evaluation was conducted during a randomised controlled trial (RCT) of the FitFor2 exercise program for pregnant women at risk for GDM. The protocol was approved by the Medical Ethics Committee of the VU University Medical Centre, Amsterdam, the Netherlands. Written informed consent was obtained from all participants. Participants were followed from 15 weeks of pregnancy till 12 weeks postpartum. More details about study design and methods of this RCT have been described elsewhere [12].

### Participant recruitment

Recruitment took place in five hospitals and 20 midwifery practices in the Netherlands, between November 2007 and April 2010. Participants were pregnant women at increased risk for GDM. Women were considered to be at increased risk for GDM if they were overweight (BMI ≥ 25) *and* had at least one of the three following characteristics: 1) history of macrosomia (offspring with a birth weight above the 97th percentile of gestational age), 2) history of GDM, or 3) first-grade relative with diabetes mellitus type 2 or if they were obese (BMI ≥ 30). Exclusion criteria included recruitment after 20 weeks of gestation; age less than 18 years; inadequate knowledge of the Dutch language; diagnosed with (gestational) diabetes mellitus before randomisation (fasting glucose >6.0 mmol/l); hypertension; alcohol abuse; drug abuse; use of medication that affects insulin secretion or insulin sensitivity; serious pulmonary, cardiac, hepatic or renal impairment; malignant disease; serious mental or physical impairment that made understanding or implementation of the study protocol/aim difficult.

### Randomisation and blinding

Eligible women were randomised into the intervention or control group. Randomisation was stratified for the hospital where participants were offered the exercise program. Within each stratum, block randomisation with blocks of four was used to ensure that each group consisted of an equal number of participants. The researcher, patients, healthcare providers and research assistant were not blinded for allocation after randomisation due to the nature of the intervention. All outcome measures were assessed by independent research assistants who were unaware of group allocation.

### Intervention

Women in the intervention group participated an exercise program twice weekly in a group during the remaining duration of their pregnancy. Each exercise session lasted for 60 minutes. The exercise sessions consisted of aerobic and strength exercises that help to control blood glucose levels. The training intensity was carefully and individually controlled. All exercise sessions were completed under the guidance and supervision of a specifically trained physiotherapist, and took place in the Department of Physiotherapy of the participating hospitals. Details of the exercise program were described previously by [Oostdam et al. 12].

Women in the control group were not offered an exercise program and received standard care from obstetricians and/or midwives. The primary task of the Dutch midwife is to closely follow the health status of the pregnant woman and her unborn child. Midwifes see their clients on average 13 times during pregnancy on an individual basis. In the Netherlands, overweight or obese women receive the same care as healthy-weight women. The control group was followed throughout the entire pregnancy period.

### Outcomes/effects

Outcome measures were maternal fasting blood glucose, insulin sensitivity, infant birth weight, and quality-adjusted life years (QALYs). Outcomes were assessed at baseline (approximately 15 weeks of pregnancy), 24 and 32 weeks of pregnancy by laboratory tests and self-administered questionnaires.

Maternal fasting blood was drawn from the antecubital vein after the participant had fasted for at least 10 hours. In this blood sample, glucose and insulin were measured. For insulin sensitivity, the homeostasis model assessment (HOMA) was calculated.

In the Netherlands, birth weight is routinely measured and recorded by the obstetrician, midwife or the nurse at delivery. Birth weight was obtained from a questionnaire filled out by the women 12 weeks postpartum.

Health-related quality of life was measured using the EuroQol-5D questionnaire [13]. Utilities were determined using the Dutch tariff [14]. Quality-adjusted life years (QALYs) were calculated by multiplying the utilities by the time spent in a given health state. Transitions between health states were linearly interpolated.

### Cost data collection and valuation

The economic evaluation was conducted from a societal perspective. Resource utilisation was assessed during pregnancy with three questionnaires at 15-, 24-, and 32-weeks of pregnancy. Direct costs included the costs of visits to healthcare providers, medication, and informal care. Indirect costs were costs related to sick leave. Costs of delivery were assessed using a questionnaire 12 weeks postpartum and were included in the direct costs.

Costs were reported in Euros and the index year was 2009 (this was the year that most data were collected). Costs were calculated by multiplying the respective units of resource use by standard costs according to Dutch Manual for Costing [15]. For visits to care providers for whom standard cost prices were not available, prices according to professional organisations were used. Medication was valued using unit prices published by Royal Dutch Society for Pharmacy [16]. Costs of absenteeism from paid work were calculated using The Friction Cost Method (FCM). The FCM assumes that costs are limited to the period necessary to replace a sick worker, the friction period. A friction period of 154 calendar days and an elasticity of 0.8 were used. The number of sick leave hours was multiplied by the cost of productivity loss per hour, based on age and gender.

### Data analysis

A power calculation was done for the primary outcome measure of maternal fasting glucose. It was determined that adequate power (>0.80) and a 5% significance level would be achieved with 80 pregnant women in both groups. The power calculation allowed for a 20% dropout rate.

The economic evaluation was performed according to the intention-to-treat principle. Due to the considerable amount of missing follow-up data, missing data were imputed using multiple imputation (MI) based on Multivariate Imputation by Chained Equations (MICE) [17]. The MI procedure was performed in SPSS 18.0, in which five complete data sets were generated. Using Rubin’s rules, effects and costs from the five complete data sets were pooled [18].

Participants with high blood glucose (>6.0 mmol/l) at baseline (n = 4) and a twin pregnancy (n = 1) were excluded from the analyses, because they did not fulfil the inclusion criteria. Insulin levels that were out-of-range, and therefore very unlikely, were coded as missing and subsequently imputed (n = 7).

The incremental cost-effectiveness ratio (ICER) is defined as the ratio of the difference in costs and the difference in effects between the intervention and standard care. ICERs were calculated for maternal fasting blood glucose, insulin sensitivity, infant birth weight and QALYs. The ICER indicates the additional investment needed to gain one additional unit of effect. For the outcome infant birth weight, the cost-effectiveness analysis was done with total societal costs including delivery costs.

Non-parametric bootstrapping with 5000 replications was used to estimate “approximate bootstrap confidence” (ABC) intervals around cost differences [19]. Bootstrapping was also used to estimate the uncertainty surrounding the incremental cost-effectiveness and cost-utility ratios (5000 replications). The bootstrapped cost-effect pairs were plotted on a cost-effectiveness plane (CE plane) [20]. The CE plane shows the difference in effect on the x-axis and the difference in costs on the y-axis. Cost-effectiveness acceptability curves (CEA curves) were also estimated. CEA curves show the probability that the intervention is cost-effective in comparison with the control treatment for a range of ceiling ratios. The ceiling ratio is defined as the amount of money society is willing to pay to gain one unit of effect [21].

Data processing was performed in SPSS 15.0. The cost-effectiveness analyses were conducted in R.

### Sensitivity analysis

In a secondary analysis, the Human Capital Approach (HCA) was used to estimate costs due to sick leave. In the HCA, total sick leave time is neither “capped” as in the FCA, nor is elasticity considered.

## Results

### Participants

A total of 425 women were invited to participate in the study of whom 121 women at risk for GDM were randomised (59 usual care group and 62 intervention group). 101 participants completed the baseline measurement and at least one follow-up measurement. The flow of the participants has been reported elsewhere [12]. The baseline characteristics of the study population are presented in Table 1. No significant differences were found between the control and intervention group for any of the variables.

**Table 1:** Baseline characteristics

**Maternal characteristics**	**Standard care Group**	**Intervention Group**	** *P* value**
	*Means ± SD* n = 52	*Means ± SD* n = 49	*Independent t-test*
Age, years	30.1 ± 4.5	30.8 ± 5.2	0.481
BMI pp^¥^, kg/m2	33.9 ± 5.6	33.0 ± 3.7	0.378
Baseline glucose, mmol/l	4.8 ± 0.5	4.7 ± 0.4	0.514
Baseline insulin sensitivity	0.07 ± 0.06	0.08 ± 0.04	0.588
Baseline utility	0.81 ± 0.18	0.83 ± 0.22	0.605
	*% (n)*	*% (n)*	*Chi-Square Test*
**Parity**			0.281
·Nulliparous	28.0 (14)	38.3 (18)	
·Multiparous	72.0 (36)	61.7 (29)	
**Race/ethnicity**			0.600
·White/Caucasian	50.0 (25)	44.7 (21)	
·Non-Caucasian	50.0 (25)	55.3 (26)	
**Educational level**			0.805
·Lower	34.7 (17)	34.0 (16)	
·Middle	34.7 (17)	40.4 (19)	
·Higher	30.6 (15)	25.5 (12)	
**Employment status**			0.282
·Employed	70.0 (35)	59.6 (28)	
·Unemployed	30.0 (15)	40.4 (19)	

Continuous variables (presented as means ± SDs) were analyzed by using an independent *t*-test; categorical variables (presented as percentages (n values)) were analyzed by using a Chi-Square test.

^¥^BMI pp: pre-pregnancy BMI, based on self-reported weight and height.

Complete healthcare cost data during pregnancy were available for 80 participants. At baseline, women with complete cost follow-up had a significantly lower pregravid BMI (mean BMI 32.9) than women without complete cost follow-up (mean BMI 35.8). For cases without complete data, missing data were imputed.

### Healthcare utilisation

The following data were derived from the complete cases only. Rates of healthcare utilisation were similar between the groups; there were no statistically significant between-group differences in terms of units of healthcare consumption. During the pregnancies of overweight women at risk for GDM, the top five most commonly used healthcare services were: midwifery (mean=8.5 , SD=5.4), gynaecology (mean=4 , SD=4.9), paramedical professional (mean=2.3 , SD=5.5), general practice (mean=2.2 , SD=2.5), hospitalisation (mean=0.7 , SD=3.4).

### Costs

Mean costs per group, mean cost differences and a 95%CI for specific cost categories are presented in Table 2. Total societal costs were €5034 (SE €744) in the intervention group and €3725 (SE €462) in the control group. However, this difference in total costs was not statistically significant. Direct costs were higher than indirect costs. Delivery costs contributed most to total direct costs, followed by hospitalisation and gynaecology costs.

**Table 2:** Cost prices (€), multiply imputed and pooled mean (SE) total costs, and mean cost differences (95%CI) during pregnancy for the intervention and the control group

	**Price € (2009)**	**Intervention group n = 49 € (SE)**	**Control group n = 52 € (SE)**	**Cost difference € (95%CI)**
General practitioner*	28	43 (7.2)	44 (13.9)	−1 (−35 ; 25)
Medical specialists*	72	31 (15)	19 (8.9)	12 (−16 ; 50)
Hospitalisation*	151 – 457	858 (392)	14 (9.7)	844 (298 ; 1955)
Occupational physician^§^	23.31	10 (4.3)	13 (4.1)	−3 (−13 ; 10)
Mental health care*	65 – 103	26 (12.5)	22 (13.2)	4 (−33 ; 38)
Paramedical professionals*^§^	34.51 – 69.34	60 (23)	108 (36)	−48 (−137 ; 24)
Dietician*	27	7 (2.3)	9 (4.1)	−2.1 (−13 ; 6)
Midwife^§^	22.60	107 (11.4)	124 (10.7)	−17 (−47 ; 14)
Obstetrician/Gynaecologist*	72	313 (51)	263 (47)	50 (−84 ; 186)
Delivery^§^	255.71 – 1047.31	1890 (242)	1568 (182)	322 (−244 ; 904)
Medications		8 (2.4)	6 (1.6)	1.9 (−3 ; 9)
**Direct costs including delivery**		3354 (491)	2189 (201)	1164 (286 ; 2431)
**Direct costs excluding delivery**		1463 (407)	622 (53)	842 (260 ; 1978)
Productivity loss FCA^¥^		1455 (533)	1536 (433)	−81 (−1306 ; 1375)
Productivity loss HCM^¥^		1498 (578)	1536 (433)	−38 (−1278 ; 1567)
Intervention^†^	21	225 (29)	0	225
**Total costs including delivery (FCA)**		5034 (744)	3725 (462)	1308 (−229 ; 3204)
**Total costs excluding delivery (FCA)**		3144 (666)	2157 (436)	986 (−424 ; 2668)

Abbreviations: €; Euros, SE; standard error, 95%CI; 95% confidence interval, FCA; friction cost approach, HCM; human capital method.

*Prices according to Dutch guidelines for costing.

^§^Prices according to professional organisation or health care provider.

^¥^Production loss per hour based on age and gender, obtained from the Dutch guidelines for costing.

^†^The cost price of the exercise program was €21 per session per participant.

Hospitalisation costs in the intervention group were significantly higher than in the control group (€844; 95%CI 298 ; 1955). This significant difference was caused by one woman who spent a few weeks in hospital because of her risk for a premature birth. No statistically significant differences in costs were observed between the intervention and control group for the other cost categories. Total costs in the intervention group were considerably higher than in the control group, but this difference was not statistically significant and was mainly caused by the already mentioned prolonged hospitalisation in the intervention group.

### Effects

Table 3 shows that no statistically significant differences were found between the intervention group and the control group on maternal fasting blood glucose (−0.021; 95%CI −0.22 ; 0.18), insulin sensitivity (HOMA) (0.006; 95%CI −0.005 ; 0.017) at 32 weeks of gestation and birth weight (156; 95%CI −83.9 ; 395.1). No statistically significant effect on QALYs (−0.005; 95%CI −0.031 ; 0.021) was observed either.

**Table 3:** Differences in pooled mean costs, effects (95%CI), and incremental cost-effect ratios (ICERs)

**Analysis**	**Sample size**		**Outcome**	**ΔC (95%CI)**	**ΔE (95%CI)**	**ICER**	**Distribution CE plane**			
	**I**	**C**		**€**			**NE**	**SE**	**SW**	**NW**
**FCA**	49	52	Fasting glucose	986 (−424 ; 2668)	−0.021 (−0.22 ; 0.18)	−46971	37.1	4.4	5.4	53.2
	49	52	IS homa	986 (−424 ; 2668)	0.006 (−0.005 ; 0.017)	162995	11.4	1	8.9	78.7
	49	52	QALY	986 (−424 ; 2668)	−0.005 (−0.031 ; 0.021)	−208558	31.4	5.4	4.7	58.4
	49	52	Birth weight	1308 (−229 ; 3204)	156 (−83.9 ; 395.1)	8.4	9.4	0.2	5.6	84.7
**HCM**	49	52	Fasting glucose	1029 (−392 ; 2830)	−0.021 (−0.22 ; 0.18)	−49008	37.4	4.3	5.5	52.9
	49	52	IS homa	1029 (−392 ; 2830)	0.006 (−0.005 ; 0.017)	170064	11.2	1	8.7	79.1
	49	52	QALY	1029 (−392 ; 2830)	−0.005 (−0.031 ; 0.021)	−217602	31.7	5.4	4.5	58.5
	49	52	Birth weight	1352 (−199 ; 3356)	156 (−83.9 ; 395.1)	8.7	9.5	0.2	5.9	84.3

Abbreviations: ΔC; costs intervention group-costs control group, ΔE; effects intervention group-effects control group, 95%CI; 95% confidence interval, ICER; incremental cost-effectiveness ratio, CE; cost-effectiveness, I; intervention group, C; control group, €; Euros, FCA; friction cost approach, HCM; human capital method.

NE; refers to the northeast quadrant of the CE plane, which indicates that the exercise program is more effective and more costly than the control group.

SE; refers to the southeast quadrant of the CE plane, which indicates that the exercise program is more effective and less costly than the control group.

SW; refers to the southwest quadrant of the CE plane, which indicates that the exercise program is less effective and less costly than the control group.

NW; refers to the northwest quadrant of the CE plane, which indicates that the exercise program is less effective and more costly than the control group.

Compliance with the intervention was low, only a small proportion of the women attended half of the training sessions (16.3%). Many women stopped exercising during the course of their pregnancy because of physical (pregnancy-related) limitations.

### Cost-effectiveness (CE) and cost-utility analyses

**Figure 1:**
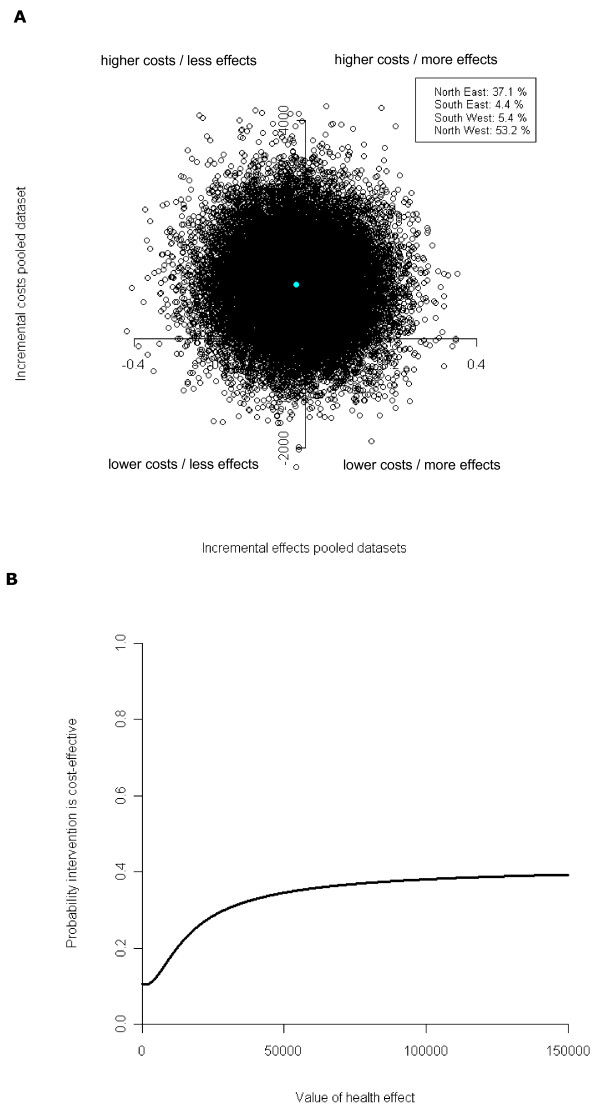
(A) CE plane and (B) CEA curve for outcome maternal fasting blood glucose (multiple imputed data).

Figure 1 shows the CE plane for the intervention versus control group for outcome of maternal fasting blood glucose at 32 weeks of gestation. The ICER for the outcome maternal fasting blood glucose was −46,971 (Table 3), meaning that to gain one point of improvement in blood glucose in the intervention group compared with the control group was associated with €46,971 higher costs. This ICER is too high to allow for meaningful interpretation. The CE plane for the outcome maternal fasting blood glucose at 32 weeks of gestation confirms the findings that there were no significant differences in total costs or blood glucose levels between the intervention and control group.

The CEA curve in Figure 1 shows that the maximum probability that the intervention group is cost-effective in comparison with the control group lies around 0.4. However, to reach this probability, society should be willing to pay a large amount of money (approximately €110,000) for an extra unit of improvement in maternal fasting blood glucose. The CE plane and ICER for the outcome insulin sensitivity showed similar results as maternal fasting blood glucose (data not shown).

The CE planes for outcomes birth weight and QALY showed that most bootstrapped cost effect pairs were situated in the northwest quadrant (84.7% and 58.4% respectively) indicating that the intervention was less effective and more costly, but not statistically significantly so.

### Sensitivity analysis

Only one woman was absent from work from the beginning of her pregnancy and exceeded the friction period. Using the HCM, the costs of lost productivity were higher than the FCA (Table 3). This resulted in larger ICERs than in the main analysis, but the conclusion of the study did not change.

## Discussion

In the present study we evaluated the cost-effectiveness of an exercise program in comparison with standard care to prevent high blood glucose levels, insulin sensitivity and high birth weight among pregnant women at risk for GDM. There were no statistically significant differences in effects, costs of health care utilisation or productivity losses between the intervention group and the control group. CE planes and CEA curves showed that the exercise program was not cost-effective compared to standard care for any of the included outcome measures.

To the best of our knowledge, our study was the first to evaluate the cost-effectiveness of an intervention to prevent GDM. A comparison with other intervention studies, therefore, is not possible. However literature has shown that costs for overweight or obese pregnant women and women at risk for GDM are higher than for women with normal weight [22-24].

Kolu [22] evaluated frequency and costs of antenatal health care visits related to risk of GDM. Visits to primary and special health care and costs of OGTT (oral glucose tolerance test) were €1553 in women at risk for GDM, which was more (10-41%) than the costs in the non-risk group. Women diagnosed with GDM had more antenatal health care visits than women without a confirmed diagnosis (17–18 visits versus 16 visits).

[Galtier-Dereure 23,^24^] investigated the cost of prenatal care in women with pregravid overweight in two separate studies. Both studies concluded that overall costs and hospital duration were higher in overweight women than in normal-weight women. Mean duration of hospitalisation and overall costs were strongly related to maternal prepregnancy weight. However, no cost estimates were presented.

In our study, we cannot make a comparison with normal weight women or women not at risk for GDM. The previous studies have not taken costs associated with a loss of productivity into account, making comparisons impossible. However, the high costs associated with hospitalisation and delivery in our study are similar to the above mentioned studies.

### Strengths and limitations

To the best of our knowledge, this was the first study to evaluate the cost-effectiveness of an exercise program for pregnant women at risk for GDM. An important strength of the current study is that the economic evaluation was conducted alongside a randomised controlled trial, where the risk of bias is limited. Another strength is that this was a pragmatic study, resembling clinical practice as much as possible.

The study also has some limitations. The first limitation is that the intervention used in our study had low compliance and showed no significant effects on fasting blood glucose, insulin, and birth weight. To examine the effect of exercise on the prevention of GDM it is important to have a good rate of compliance with the intervention. Perhaps with another type of intervention, such as a counselling intervention, a better compliance and engagement with physical activity and exercise can be achieved for this group of obese pregnant women. Another limitation is the considerable loss to follow-up data, which may have reduced the reliability of the results. To account for this, missing values were imputed by using the MICE procedure. Multiple imputation takes into account the uncertainty associated with imputing missing values and is recommended to impute missing cost data [25]. However, missing values might have compromised validity of or findings. In addition, the RCT was underpowered to detect cost differences, because the power calculation was based on maternal fasting blood glucose. Cost data usually follow a highly skewed distribution, implying a need for larger sample sizes in cost/effectiveness studies as compared to effectiveness studies, [25] which may be considered unfeasible or even unethical.

Self-reported cost data were collected every 10 weeks, which may have introduced recall bias. However, this method was used in both the intervention and control group, so it is unlikely that it will have influenced our results. Furthermore, van den Brink et al. showed that a cost questionnaire with structured closed questions might replace a cost diary for recall periods up to six months [26].

## Conclusion

The exercise program for pregnant women at risk for GDM evaluated in the present study was not found to be cost-effective compared to usual care to prevent high blood glucose levels, insulin sensitivity and high infant birth weight. Factors such as low compliance, and lack of power may explain these results. We feel that other types of intervention may be necessary to engage this target group in physical activity and exercise. Furthermore, to reduce costs in women at risk for GDM, the focus should be on interventions that may reduce delivery costs and productivity losses. Based on these results, implementation of this exercise program in the prevention of GDM cannot be recommended.

## Abbreviations

GDM, Gestational diabetes mellitus; RCT, Randomised controlled trial; QALY, Quality-adjusted life-years; T2D, Type 2 diabetes; CE, Cost-effectiveness; HOMA, Homeostasis model assessment; FCM, Friction Cost Method; MICE, Multivariate Imputation by Chained Equations; HCA, Human Capital Approach; ICER, Incremental cost-effectiveness ratio; ABC, Approximate bootstrap confidence; CE Plane, Cost-effectiveness plane; CEA Curves, Cost-effectiveness acceptability curves; OGTT, Oral glucose tolerance test.

## Competing interests

The author’s declare that they have no competing interests.

## Authors’ contributions

NO was responsible for data collection, performed statistical analyses (with contributions of JB) and drafted the first version of the manuscript. MvP, MW, EE, WvM contributed to the conception of the trial. NO, JB, MvP thoroughly revised the manuscript and aided in data interpretation. All authors read and corrected draft versions of the manuscript and approved the final manuscript.

## Pre-publication history

The pre-publication history for this paper can be accessed here:

http://www.biomedcentral.com/1471-2393/12/64/prepub
